# Novel NAPRT specific antibody identifies small cell lung cancer and neuronal cancers as promising clinical indications for a NAMPT inhibitor/niacin co-administration strategy

**DOI:** 10.18632/oncotarget.20840

**Published:** 2017-09-12

**Authors:** Jonathan Cole, Marie-Christine Guiot, Michel Gravel, Cynthia Bernier, Gordon C. Shore, Anne Roulston

**Affiliations:** ^1^ Laboratory for Therapeutic Development, Rosalind and Morris Goodman Cancer Research Centre, and Department Biochemistry, McGill University, Montreal, QC, Canada; ^2^ Department of Pathology, Montreal Neurological Hospital, Montreal, QC, Canada

**Keywords:** NAMPT, NAPRT, biomarker, NAMPT inhibitor, NAD^+^ biosynthesis

## Abstract

Tumor cells are particularly dependent on NAD^+^ due to higher rates of metabolism, DNA synthesis and repair. Nicotinamide phosphoribosyltransferase inhibitors (NAMPTis) inhibit NAD^+^ biosynthesis and represent promising new anti-cancer agents. However, clinical efficacy has been limited by toxicities demonstrating the need for drug combinations to broaden the therapeutic index. One potential combination involves niacin/NAMPTi co-administration. Niacin can rescue NAD^+^ biosynthesis through a parallel pathway that depends on nicotinic acid phosphoribosyltransferase (NAPRT) expression. Most normal tissues express NAPRT while a significant proportion of malignant cells do not, providing a possible selection marker for patients to achieve NAMPTi efficacy while minimizing toxicities.

Here we identify and validate a novel highly NAPRT-specific monoclonal antibody (3C6D2) that detects functional NAPRT in paraffin embedded tissue sections by immunohistochemistry (IHC). NAPRT detection by 3C6D2 coincides with the ability of niacin to rescue cells from NAMPTi induced cytotoxicity in cell lines and animal xenograft models. 3C6D2 binds to an epitope that is unique to NAPRT among phosphoribosyltransferases. In a series of primary tumor samples from lung and brain cancer patients, we demonstrate that >70 % of human small cell lung carcinomas, glioblastomas and oligodendrogliomas lack NAPRT identifying them as potentially suitable indications for the NAMPT/niacin combination.

## INTRODUCTION

Nicotinamide adenine dinucleotide (NAD^+^) plays a crucial role in cancer cell metabolism and is the substrate for important cancer related enzymes such as ADP-ribose transferases, including poly-ADP-ribose polymerases (PARPs), sirtuins and cyclic ADP (cADP) ribose synthases. These enzymes are important in DNA repair, G-protein coupled receptor signaling, calcium homeostasis, transcriptional regulation, and ultimately cancer cell survival [1, 2]. Interfering with NAD^+^ biosynthesis as a therapeutic strategy for cancer holds great promise. Cancer cells are particularly dependent on NAD^+^ to maintain rapid growth, DNA replication, and repair [2–5]. When NAD^+^ is depleted, intracellular ATP depletion follows soon after and cells die by autophagy, parthanatos, or by oncosis (reviewed in [6]). In mammalian cells, NAD^+^ is synthesized by two parallel routes: the primary pathway is via nicotinamide phosphoribosyltransferase (NAMPT) that derives NAD^+^ from the pre-cursor, nicotinamide [2, 7]; the secondary Preiss-Handler pathway, that operates in parallel, derives NAD^+^ from nicotinic acid (niacin), via nicotinic acid phosphoribosyltransferase (NAPRT) [8, 9]. NAMPT is the key biosynthetic enzyme involved in NAD^+^ generation and recycling in cells and is a compelling anti-cancer drug target.

Within the last decade, two distinct chemical classes of NAMPT inhibitors (NAMPTis) have been in clinical trials as single agent therapies but unfortunately, did not show efficacy [10, 11]. The dose limiting toxicities observed in humans included gastrointestinal effects, thrombocytopenia, skin rash, and lymphopenia, likely reflecting the systemic on-target effects of NAMPT inhibitory activity [10, 12, 13]. These side-effects are reminiscent of the symptoms of pellagra, a condition caused by niacin deficiency [14]. Recently, several studies have proposed a novel means of mitigating NAMPTi-mediated systemic toxicities through a drug combination strategy involving NAMPTi and niacin co-administration [15–21]. Early studies with NAMPTis demonstrated that cellular NAD^+^ depletion and cytotoxicity of cancer cells can be rescued by addition of exogenous niacin [22]. Cell death rescue through this pathway was determined to be dependent on the presence of NAPRT expression [16, 17, 23]. NAPRT is expressed in the majority of healthy tissues in mammals but a significant proportion of solid tumors, sarcomas, lymphomas, and glioblastomas are NAPRT deficient [18]. Therefore, one potential strategy to widen the therapeutic index of NAMPTis is to protect NAPRT positive healthy tissue but not NAPRT deficient tumor tissue by niacin co-administration [11][24]. This will safely enable the administration of clinically effective doses of NAMPTis in cases where patient tumors do not express NAPRT. Studies in mice demonstrate that co-administration of niacin with lethal doses of NAMPTi rescued mortality and other toxicities such as thrombocytopenia and lymphopenia [16, 17, 19]. Niacin co-administration also minimized histologic signs of toxicity to testis, spleen, and lymphoid tissue, and reversed the toxicity to kidney and gastrointestinal tissues [17]. Furthermore, niacin co-administration did not diminish anti-tumor activity in xenograft models when administered in the appropriate doses and enhanced anti-tumor activity could be achieved due to the increased tolerance of NAMPTi in the presence of niacin [16, 17].

To effectively select patients who may benefit from the niacin/ NAMPTi strategy, a suitable test to detect functional NAPRT is needed. According to The Human Protein Atlas, NAPRT mRNA is expressed in most tissues at low levels, however, protein levels are highly variable across different tissues [24]. Work by Shames et al. [23] demonstrated that *NAPRT* gene expression is regulated by promoter hypermethylation causing suppression of *NAPRT* expression in 5-65 % of various solid tumor types. More recent data characterizes multiple levels of *NAPRT* gene regulation including promoter mutation and methylation. The existence of multiple alternatively spliced *NAPRT* transcripts, some of which are predicted to translate into proteins lacking the enzymatically active domains of the protein [25, 26], have also been identified. Therefore, the development of a reliable test for NAPRT protein expression that predicts enzymatic activity, requires a highly specific antibody recognizing active NAPRT for selection of patients potentially responsive to the niacin/NAMPTi combination strategy.

Here we demonstrate the generation and characterization of a highly specific NAPRT monoclonal antibody that detects functionally active human NAPRT. The epitope to which the 3C6D2 antibody binds is on the enzyme surface and allows for sensitive and quantitative NAPRT protein detection in formalin fixed paraffin embedded (FFPE). This antibody stains FFPE tissue more specifically and at lower concentrations than four commercially available NAPRT antibodies. Using this antibody and IHC staining on tissue biopsy samples, we examine NAPRT expression in a series of primary human lung and brain cancer subtypes and in corresponding normal tissue. We demonstrate that >70 % of small cell lung carcinoma (SCLC) tumors, glioblastomas, oligodendrogliomas and astrocytomas lack NAPRT identifying them as suitable indications for the NAMPT/niacin combination strategy.

## RESULTS

We developed a monoclonal antibody by immunizing mice with a protein fragment of human NAPRT corresponding to amino acids 256-515. This peptide region of NAPRT was predicted to be immunogenic and included portions of the enzymatically active domains (26). Sera from immunized mice were screened for NAPRT reactivity by immunocytochemistry (ICC) using FFPE samples prepared from NAPRT positive cell lines and by ELISA using the peptide immunogen. Hybridoma clones were generated from mice with positive staining sera and sub-cloned to produce clone 3C6D2, expressing an antibody with high specificity for NAPRT by ICC and ELISA.

### Monoclonal antibody 3C6D2 detects NAPRT

**Figure 1:**
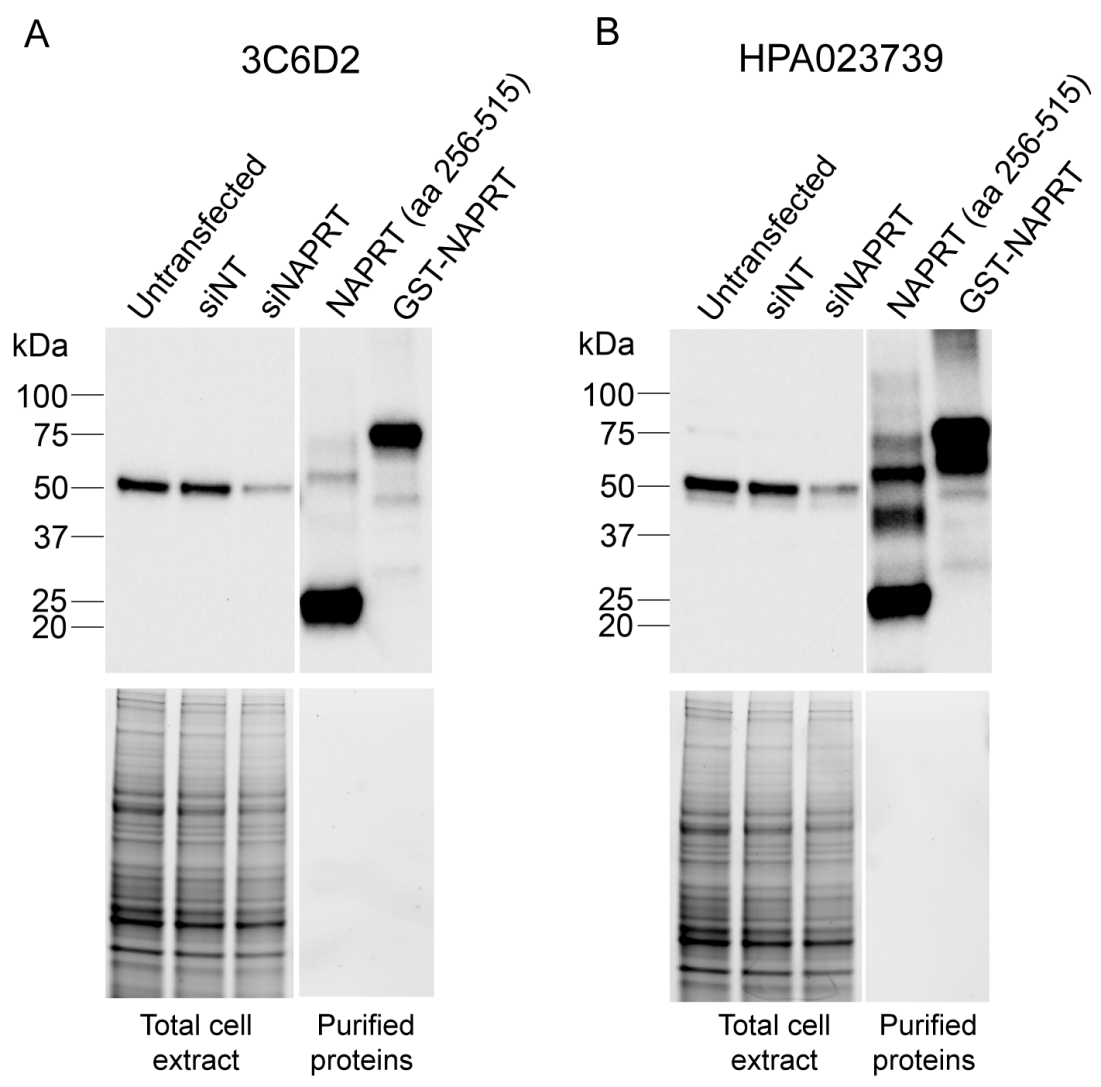
Monoclonal antibody 3C6D2 is highly specific for NAPRT protein H1299 cells were transfected with either a non-targeting siRNA (siNT), an NAPRT-targeting siRNA (siNAPRT) as indicated for 72 h and cell lysates harvested. 10 μg of whole cell lysates from siRNA transfected cells or 2 ng of purified NAPRT immunization fragment (aa 256-515) or recombinant GST-NAPRT were resolved on BioRad stain-free polyacrylamide gels and detected by immunoblotting with (**A**) the 3C6D2 monoclonal antibody or (**B**) a commercially available polyclonal NAPRT specific antibody (HPA023739). Total protein was visualized on the protein containing nitrocellulose membrane using a fluorescence detection imager.

Immunoblot analyses of H1299 cell extracts indicate that monoclonal antibody 3C6D2 is highly specific for NAPRT as it recognizes a single band: the predicted 55 kDa full length protein from total cell extracts (Figure 1A). This band intensity is reduced following siRNA knockdown with a human *NAPRT*-targeted siRNA in H1299 cells demonstrating that it corresponds to NAPRT. As expected, the 3C6D2 antibody also recognizes the recombinant full length protein and the fragment of NAPRT used in the immunization protocol (Figure 1A). Detection of human NAPRT by 3C6D2 using the same protein samples is compared to a commercially available NAPRT specific polyclonal antibody HPA023739 (Sigma) (Figure 1B) derived from rabbit immunizations with a human NAPRT peptide (amino acids 285-420). HPA023739 is a validated antibody used to characterize NAPRT tissue distribution in The Human Protein Atlas [24, 27]. Interestingly, HPA023739 detects several additional bands by immunoblotting of the recombinant proteins (compare Figure 1A and 1B) indicating that the protein recognition of the two antibodies is similar but not identical. Together, these results demonstrate that the 3C6D2 monoclonal antibody recognizes NAPRT with very high specificity.

### Absence of full length NAPRT expression coincides with cytotoxicity in the presence of niacin and NAMPT inhibitor

**Figure 2:**
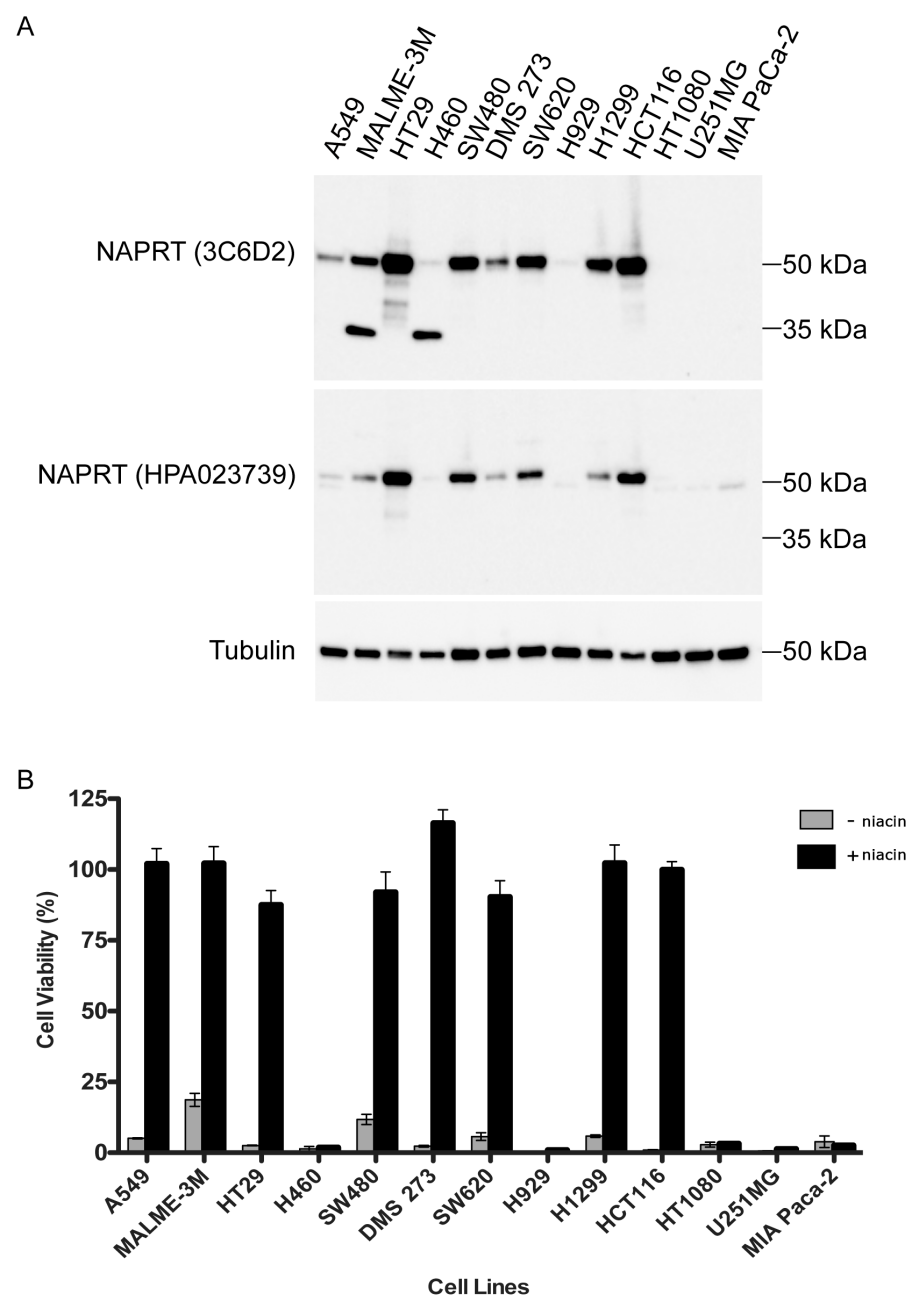
Detection of functional NAPRT by 3C6D2 in human tumor cell lines (**A**) NAPRT expression levels were determined in a panel of cancer cell lines by immunoblotting using monoclonal antibody 3C6D2 or the polyclonal antibody HPA023739. Tubulin staining was used as a protein loading control. (**B**) Functional NAPRT activity was assessed in cells treated with a lethal dose of the NAMPT inhibitor GMX1778 (50 nM) in the presence or absence of exogenous niacin (1 μM) for 72 h. Viability was determined using the Cell-Titer Glo assay and is expressed as the mean percentage viability relative to cells treated without GMX1778. Error bars represent the SD (n=6 replicates).

As previously demonstrated, when functional NAPRT is present, cells treated with niacin are rescued from cell death induced by NAMPT inhibitors [16, 17, 22]. To evaluate the expression of NAPRT isoforms detected by 3C6D2, a series of protein extracts from cancer cell lines from different tissues of origin were subject to immunoblotting with this antibody (Figure 2A). The 3C6D2 antibody detects full length NAPRT in most cell lines with the exception of H460 (non-small cell lung), H929 (multiple myeloma), HT1080 (osteosarcoma), U251MG (glioblastoma) and MIA PaCa-2 (pancreatic) cells. A549 (non-small cell lung) cells express lower levels but clearly detectable full length NAPRT. Interestingly, MALME-3M (melanoma) and H460 cells have strong expression of an apparent truncated NAPRT protein of approximately 35 kDa detected with 3C6D2 but not the HPA023739 polyclonal antibody. Immunoblotting was used to evaluate three other commercially available monoclonal antibodies: CLO366, 4A9 and 5B0 at concentrations up to 4 μg/mL. CLO366 was the only antibody to generate a signal on the blot; the band detected at ~55 kDa corresponds to NAPRT (Supplementary Figure 1).

To determine whether the NAPRT protein detected is functionally active, the same series of cell lines were treated with a cytotoxic dose of GMX1778, a high affinity NAMPT inhibitor [16, 19, 28], in the presence or absence of niacin (Figure 2B). Cell viability was measured using an ATP-based cell viability readout. Previous data demonstrated that GMX1778 concentrations required to reduce intracellular ATP correlate with loss of cell viability measured by cell lysis [16]. Treatment with GMX1778 alone for 72 hours is sufficient to reduce the ATP levels of all cell lines to <20 %. However, treatment with GMX1778 + niacin fully rescues the ATP levels of all cell lines expressing full length NAPRT but does not rescue any cell lines lacking NAPRT expression. Viability of the A549 cell line is also rescued, suggesting that even low expression levels of NAPRT are sufficient to rescue from NAMPTi induced cell death. Interestingly, the H460 cell line, which expresses only the 35 kDa NAPRT fragment, is not rescued suggesting that the smaller NAPRT isoform is not functionally active. To be certain that the 35 kDa band detected by 3C6D2 represents an isoform or fragment of NAPRT and not an unrelated protein, H460 cells were transfected with a siRNA targeting human NAPRT (Supplementary Figure 2). Immunoblot analyses indicates that protein expression levels of the 35 kDa isoform are reduced by the NAPRT specific siRNA relative to a non-targeting siRNA, demonstrating that the 35 kDa protein represents an isoform or fragment of NAPRT. The fact that 3C6D2 but not HPA023739 can detect the 35 kDa NAPRT protein fragment suggests that this protein isoform or fragment contains the C-terminal portion of NAPRT and that the epitope to which 3C6D2 binds lies within amino acids 420-515 of NAPRT.

### The 3C6D2 antibody detects NAPRT in FFPE tumor tissue samples

**Figure 3:**
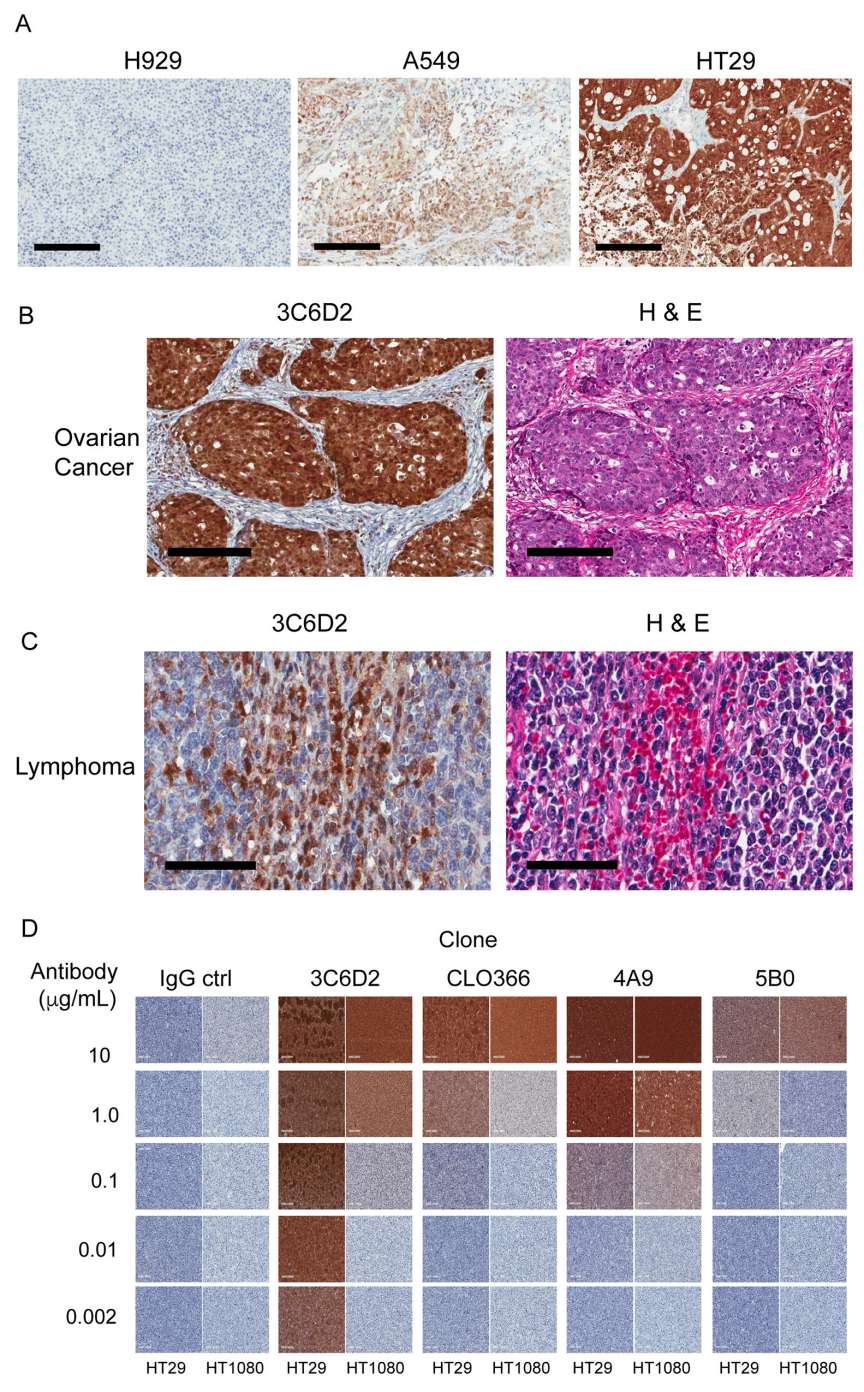
Immuno-histochemical detection of NAPRT expression from FFPE human xenograft, histology and cell line samples (**A**) NAPRT expression in three FFPE embedded xenograft tumors from cell lines: H929 (multiple myeloma), A549 (non-small cell lung) and HT29 (colon). Xenograft slices were stained with 3C6D2 and CAT hematoxylin. Scale bars represent 200 μm. (**B** and **C**) Examples of NAPRT expression from FFPE human tumor samples from (B) ovarian cancer and (C) lymphoma. Adjacent slices of tumor tissue were stained with 3C6D2 and CAT hematoxylin counter stain (left) and hematoxylin and eosin staining (H&E) (right). Scale bars represent 60 μm (lymphoma) or 200 μm (ovarian cancer). (**D**) FFPE cell pellets from NAPRT positive HT29 and NAPRT negative HT1080 cells were stained with the concentration range of various commercially available NAPRT monoclonal antibodies as indicated and CAT hematoxylin counter stained. IgG1 and IgG2α were used as a negative staining control.

To accurately evaluate the lack of NAPRT expression in primary tumor tissue while distinguishing it from surrounding normal tissue and endothelial tissue, we examined FFPE samples by IHC. To test the feasibility of 3C6D2 antigen detection with this technique, cell pellets from the panel of cancer cell lines evaluated in Figure 2 were fixed and embedded in paraffin wax consistent with the way tumor biopsy samples are prepared. Tumor sections underwent optimized ICC staining that includes a heat induced epitope retrieval step and the lowest concentration of the 3C6D2 antibody (1 ng/mL) required to detect NAPRT in A549 cells. The results demonstrate that all cell lines expressing full length NAPRT (as detected by immunoblot: Figure 2A) also stain positively for NAPRT by ICC (Table 1). The intensity of staining coincides directly with the levels of NAPRT expression determined by immunoblot (Figure 2A); three examples of this are illustrated in Figure 3A. Importantly, H460 cells that express the shorter, 35 kDa NAPRT isoform, stain negative for NAPRT by ICC (Supplementary Figure 2), indicating that the smaller non-functional NAPRT isoform is not detected with 3C6D2 in FFPE tissue samples. To determine whether 3C6D2 can detect NAPRT in human FFPE biopsy specimens, tissue sections from two different tumor types were stained. The 3C6D2 antibody detected NAPRT in patient derived primary tumor sections and clearly distinguishes positive and negative staining areas (Figure 3). In Figure 3B, NAPRT expression is detected in ovarian carcinoma cells but not in the surrounding stroma. Previous work by Shames et. al. has demonstrated that 90 % of ovarian cancer cell lines are NAPRT positive [23]. Figure 3C depicts a core biopsy of lymphoma, where tumor cells are NAPRT negative but infiltrating lymphocytes are strongly positive. This is consistent with data from lymphoma cell lines and tissue sections indicating that 60-75 % of lymphomas/leukemias are NAPRT negative [16, 23]. As observed in these tissue slices and cancer cell lines (Figure 3), NAPRT stains strongly in the nucleus but is also present in the cytoplasm of cells. These results are consistent with the staining pattern observed in The Human Protein Atlas with the NAPRT-specific HPA023739 antibody [27].

**Table 1:** Summary of NAPRT detection and function in a panel of cell lines

Cell line	Tumor type	Functional NAPRT*	NAPRT detection by Immunoblot**	NAPRT detection by ICC
A549	NSCL	+	+	+
MALME-3M	Melanoma	+	+	+
HT-29	Colon	+	+	+
H460	NSCL	-	-	-
SW480	Colon	+	+	+
DMS 273	SCL	+	+	+
SW620	Colon	+	+	+
H929	Multiple myeloma	-	-	-
HCT116	Colon	+	+	+
HT1080	Fibrosarcoma	-	-	-
U251MG	Glioblastoma	-	-	-
MIA Paca-2	Pancreatic	-	-	-

*Determined by niacin rescue.

** Presence of a ~50-55 kDa protein band.

The specificity of 3C6D2 antibody staining was compared to three other commercially available murine monoclonal NAPRT antibodies in an ICC assay using as test samples, FFPE embedded HT29 (NAPRT positive) and HT1080 (NAPRT negative) cell pellets (Figure 3D). Antibody clone 3C6D2 was able to clearly distinguish between NAPRT positive and negative samples at an antibody concentration range of 0.002 to 1.0 μg/mL (lower antibody concentrations were not tested). Commercial antibody clone CLO366 could distinguish positive and negative samples at 1.0 μg/mL only, whereas each of the other two monoclonal antibodies could not readily distinguish between NAPRT positive and negative samples at all concentrations tested. 3C6D2 was also compared to the polyclonal antibody HPA023739 by ICC staining in the same cell types (Supplementary Figure 3). Distinguishing NAPRT positive and negative samples with HPA023739 was only evident at 0.1 μg/mL; this is in contrast to the 3-log detection range of 3C6D2. These results indicate that compared to the commercially available antibodies, 3C6D2 has the highest specificity.

### 3C6D2 binds to an eight amino acid epitope near the dimerization domain of NAPRT

**Figure 4:**
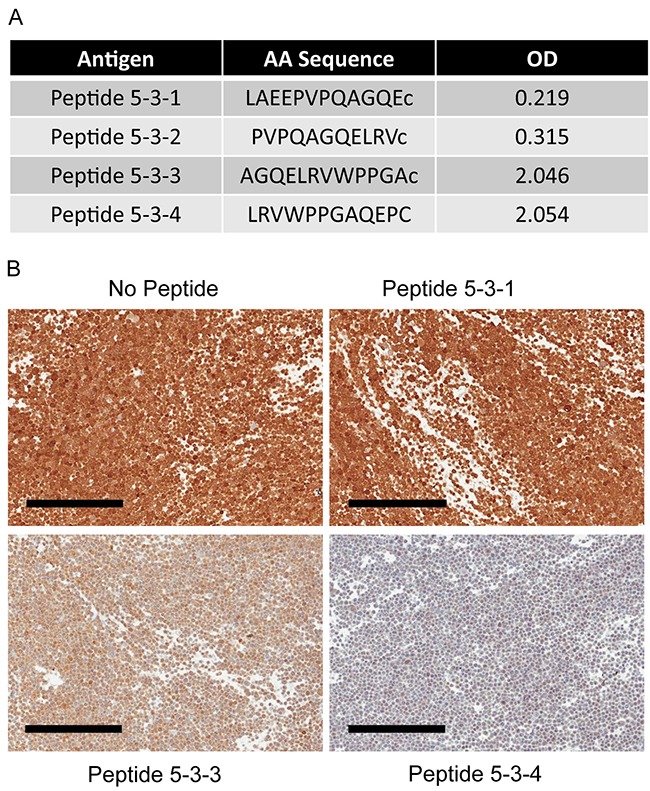
Epitope mapping of 3C6D2 antibody by ELISA and IHC staining with epitope peptide competition (**A**) ELISA assay reactivity for four peptides near the 3C6D2 antibody epitope on NAPRT. OD, optical density (**B**) ICC staining of HT-29 cell pellets with 3C6D2 in the presence or absence of a 200-fold molar excess of epitope containing peptides as indicated. Scale bars, 200 μm.

The epitope to which 3C6D2 binds within NAPRT was mapped using sequentially truncated human NAPRT peptides in an ELISA competition assay. The epitope mapped to a stretch of 8 amino acids common to two overlapping peptides: LRVWPPGA corresponding to amino acids 452 to 459 of human NAPRT (isoform 1) (Figure 4A). To demonstrate the specificity of 3C6D2 and reactivity to this epitope by ICC, HT29 FFPE cell pellets were stained in the presence or absence of excess peptides. Both peptides containing the 8 amino acid epitope (peptide 5-3-3 and 5-3-4) competed for 3C6D2 binding to the NAPRT positive sample stained by ICC, confirming the epitope to which this antibody binds in FFPE samples (Figure 4B). The epitope is situated between β18 and β19 β-sheets of human NAPRT [29], within the “open sandwich” structure and within a region distinct from other phosphoribosyltransferases. These amino acids face outward but are near to a protein dimerization domain. As NAPRT dimerization is required for enzyme function [30, 31], these residues could be important for NAPRT activity even though they are distant from the catalytic regions.

### A high frequency of brain tumors and small cell lung carcinoma are NAPRT negative

**Figure 5:**
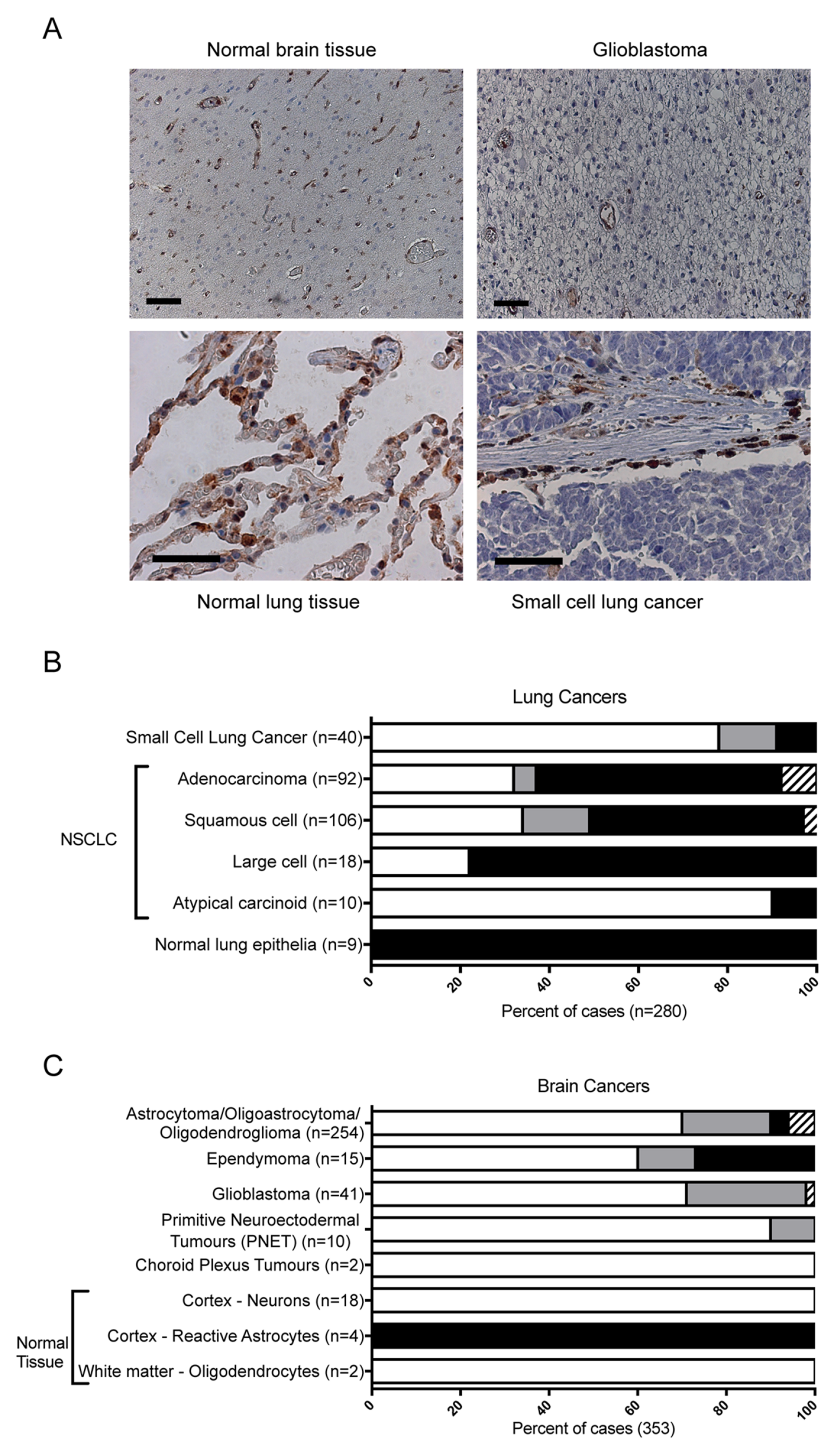
Frequency of NAPRT staining in human lung and brain cancers (**A**) A representative comparison of NAPRT expression levels in human tumor-adjacent normal brain tissue (upper left) and glioblastoma tumor tissue (upper right) or in normal lung (lower left) compared to small cell lung carcinoma (lower right). Each sample was stained with 3C6D2 and CAT hematoxylin. Scale bars: 100 μm (brain) and 40 μm (lung). (**B**) Lung or (**C**) brain cancer tumor micro arrays (TMAs) were stained with 3C6D2/CAT hematoxylin by IHC to determine expression levels of NAPRT protein. The frequency NAPRT positive cancerous cells within the tumor sample was scored as 0-10 % (white), 11-90 % (grey) or 91-100 % (black) positively stained. Samples scored as equivocal (hatched bars) could not be evaluated. The results are indicated as the percentage of total cases examined within a tumor sub-type. n = the number of different tumor cases evaluated.

To understand the frequency of lung and brain tumor sub-types that are NAPRT negative and therefore represent patients likely to benefit from the niacin + NAMPT inhibitor combination, several commercially available tumor microarrays were stained with the 3C6D2 antibody and the proportion of cells within each sample scored for NAPRT expression. A slide containing NAPRT negative (H929), and positive (A549 and HT29) cell line samples (Figure 3A) were included with each staining batch as controls. In the evaluation of tumor samples, cells were scored positive for NAPRT when either nuclear or cytosolic staining was observed; often, the staining appeared as both nuclear and cytosolic. Figure 5A (upper left) depicts healthy brain cortex with endothelial cells of the blood vessels staining positive for NAPRT; Figure 5A (upper right) depicts a glioblastoma tissue section where glioblastoma cells stain NAPRT negative but endothelial cells within the tumor stain NAPRT positive. Endothelial cells therefore serve as an internal positive staining control for any tissue that stains negative. Figure 5A (lower left) depicts normal human lung epithelia staining strongly positive (++) for NAPRT. Figure 5A (lower right) represents a small cell lung cancer tissue section where tumor cells are NAPRT negative and infiltrating inflammatory cells present in the stroma are NAPRT positive.

Tissue microarrays (TMAs) of various brain and lung cancer samples were scored for NAPRT staining. Individual cells in each tumor sample were scored as negative or positive +, ++ or +++, depending on the intensity of NAPRT staining. All levels of positive staining (+, ++ or +++) were considered positive based on the fact that low level NAPRT expression in cancer cell lines is sufficient to rescue cell death from NAMPTi inhibition with niacin. In addition, the proportion of tumor cells within the tumor field of view was scored as a percentage and grouped into categories: 0-10 %, 11-90 % or 91-100 % NAPRT positive and is illustrated graphically. The proportion of NAPRT positively staining cells within tumor sections across multiple samples is summarized according to tumor sub-type of lung (Figure 5B) or brain (Figure 5C) tumors. For all tumor types examined, tumors were most frequently either 0-10 % NAPRT positive (considered negative) or 91-100 % positive. Of note, within the NAPRT negative staining classification in all tumor types, 90 % of tumor samples were homogeneously NAPRT negative and only 10 % of this subgroup had heterogeneously staining tumors with infrequent NAPRT positive cells (ie 1-10 % of the tumor cells NAPRT positive). Importantly, this indicates that most often tumor tissue was highly homogeneous and that NAPRT tumor status is clearly either positive or negative.

Strikingly, among the various lung cancer subtypes, >75 % of small cell lung carcinoma and atypical carcinoid tumor cases were NAPRT negative (Figure 5B), suggesting these indications would be well suited to the niacin + NAMPTi combination strategy. Among the remaining non-small cell lung cancer subtypes examined, 20-35 % of cases scored negative for NAPRT expression (0-10 % NAPRT positive) consistent with a previous report scored by IHC from paraffin embedded tissue using a different commercially available antibody [23]. All normal lung epithelial tissue stained positive for NAPRT and is consistent with data in The Human Protein Atlas [24].

Among the brain tumor subtypes examined, the majority of cases (> 60 %) were NAPRT negative, including glioblastomas, astrocytomas, and dendrogliomas, that were together, >70 % negative (Figure 5C), suggesting that patients with these tumor types may also benefit from the niacin + NAMPTi combination strategy. However, in non-tumorigenic brain cortex, neurons were NAPRT negative while reactive astrocytes seen in some of the samples were positive (data not shown). In samples of non-tumorigenic white matter, oligodendrocytes were also NAPRT negative (Figure 5C). These results on a small number of samples suggest that further study is warranted to evaluate different types of normal neuronal cells within the brain.

## DISCUSSION

Successful implementation of the strategy to widen the therapeutic index of NAMPT inhibitors by co-administration with niacin, will depend on the ability to correctly detect functional NAPRT in tumor tissue and on the ability of normal tissue to be protected by niacin administration. NAPRT converts niacin to nicotinic acid mononucleotide (NAMN) [30], which is then converted to nicotinic acid dinucleotide (NAAD+) and NAD^+^ by NMNATs and NAD synthetase 1 respectively, to complete the Preiss-Handler pathway [1]. In a previous study, a panel of 77 cancer cell lines demonstrated that in all but one cell line, NAMN, but not niacin, could rescue cytotoxicity induced by GMX1778 indicating that the Preiss-Handler pathway to NAD^+^ is intact downstream of NAPRT and that rescue depends solely on the expression of NAPRT [16]. Sensitivity to NAMPTis as single agents correlates with decreased NAMPT but not NAPRT expression, whereas, sensitivity to the niacin + NAMPTi drug combination correlates with decreased NAPRT expression [16, 17, 23]. Accurately predicting functional NAPRT expression in tumor cells will therefore be crucial for the selection of patients for this combination strategy.

Robust methods used to stratify patients for personalized therapies in oncology are typically based on one of several techniques: IHC for protein detection, fluorescence in situ hybridization (FISH) and quantitative PCR (qPCR) for mRNA expression, gene amplifications/deletions or mutations, and more recently, methylation-specific qPCR techniques can quantify epigenetic changes in gene regulation (reviewed in [32]). To use NAPRT as a stratification biomarker, the most effective method for NAPRT detection will likely involve IHC to detect protein expression, for two reasons: First, to allow the discrimination of NAPRT protein present in tumor cells from tumor infiltrating endothelial and immune cells and surrounding normal tissue. Second, protein expression will be the most direct way to quantify functional NAPRT in view of limitations to the evaluation of genomic DNA, mRNA, or epigenetic expression in the case of *NAPRT* as discussed below.

Recent studies have characterized *NAPRT* gene regulation and expression patterns including *NAPRT* splice variants in normal and cancer tissues [18]. Homozygous gene deletions of *NAPRT* are rare and loss of heterozygosity occurs in less than 20 % of cancer tissues according to The Cancer Genome Atlas (TCGA) [23]. Similarly, despite search efforts, there are no genetic mutations predicted to give rise to functionally inactive NAPRT protein in tumor cells [25, 26]. These data suggest that lack of functional NAPRT expression cannot be predicted based on features of genomic DNA. However, Shames et al. determined that *NAPRT* gene expression is suppressed by promoter methylation at CpG islands [23]. This gives rise to the possibility of using a quantitative methylation-specific PCR method to predict lack of *NAPRT* expression in tumor tissue. This method was demonstrated to be predictive of niacin rescue in homogeneous tissues such as cell lines [23]. In another study, tumors with mutated IDH1 gave rise to increased CpG island methylation at the *NAPRT* promoter, resulting in decreased *NAPRT* expression [33]. However, in patient tumor tissue samples, it is not clear whether promoter methylation will consistently suppresses NAPRT protein expression sufficiently to prevent niacin rescue. Our *in vitro* results indicate that even low levels of NAPRT protein expression by ICC are sufficient to fully rescue cell survival in the presence of niacin (eg. A549 cells, Figure 2B). For this reason, indirect methods such as IDH1 mutation detection and *NAPRT* promoter methylation are accompanied by limitations to reliable NAPRT protein detection. Therefore, a highly specific and sensitive antibody against NAPRT protein such as 3C6D2 may be better suited for predicting susceptibility of tumor tissue to the niacin/NAMPTi combination in complex and heterogeneous patient tumor samples.

The quantification of *NAPRT* mRNA also presents limitations for predicting the presence of functional NAPRT protein as inconsistencies between the levels of mRNA and protein expression have been reported [25, 26], suggesting additional levels of protein regulation. Duarte-Pereira et al. have identified multiple putative *NAPRT* splice isoforms, some of which are predicted to give rise to smaller inactive protein fragments [25, 26]. The epitope to which 3C6D2 binds (amino acids 452-459) is found within exon 11 near a protein dimerization domain of NAPRT. The 35 kDa protein detected in H460 and MALME-3M cells may represent an example of the product of a splice isoform that includes exon 11, but lacks exons coding for the catalytically active regions of NAPRT [25] and is therefore non-functional. Although 3C6D2 recognizes this smaller inactive protein product by immunoblot, importantly, it does not recognize it by ICC, which could otherwise lead to a false positive result. Similarly, several polymorphisms within *NAPRT* have been identified [25], although none occur within the region encoding the 3C6D2 binding epitope. This eliminates the possibility of potentially false negative scores resulting from functionally active *NAPRT* polymorphisms not being detected by the 3C6D2 antibody. Compared with other commercially available monoclonal antibodies, 3C6D2 is able to distinguish positive and negative staining over at least a 3 log concentration range indicating that it is highly specific. Collectively, our results strongly support 3C6D2 as an extremely useful reagent that warrants its clinical use in a companion theranostic test for accurate implementation of patient stratification for the niacin/NAMPTi therapeutic strategy.

Here we have used 3C6D2 to detect NAPRT levels by IHC in multiple FFPE tumor tissue samples from patients with various lung and brain tumors. In the series of tumor tissue cases examined to date, it appears that tumor samples are most frequently either NAPRT positive (90-100 % positive), or negative (0-10 % positive), with less than 15 % of samples being partially positive (11-90 %) in tumor tissue other than very heterogeneous tumors such as glioblastoma. Within the negatively scoring group (0-10 % positive tumor cells), greater than 90 % of the tumor samples were homogeneous with all tumor cells scoring NAPRT negative, the remaining proportion of this group were heterogeneous tumors in which up to 10 % of the tumor cells were NAPRT positive. This predominantly binary readout facilitates assay interpretation, scoring, patient stratification, and provides the opportunity for a primarily homogeneous anti-tumor response. However, the possibility remains that patients with heterogeneous tumors (predominantly NAPRT negative tumor cells) may respond well to therapy initially, but residual NAPRT positive tumor cells could later persist and regrow in the presence of NAMPTi and niacin therapy. In anticipation of resistance in patients with heterogeneously staining tumors, an accurate theranostic test could be used to stratify patients for additional treatment with an unrelated agent. Interestingly, among all lung tumor samples tested, SCLC and atypical carcinoid tumor subtypes had the highest proportion of NAPRT negative tumors (>75 %). Similarly, >70 % of glioblastomas, oligodendrogliomas and astrocytomas were NAPRT negative, identifying these as indications highly suitable for the NAMPTi/niacin combination therapy. Of the remaining lung indications, 25-30 % NAPRT were negative suggesting that a theranostic test would be necessary to identify patients suitable for the niacin/NAMPTi combination.

In addition to its potential use in theranostic tests for NAMPTi therapeutic strategies, the highly specific 3C6D2 antibody will serve as an important tool to evaluate NAPRT status within normal, complex organs such as the brain. The most extensive description of NAPRT protein expression patterns in normal tissues exists in The Human Protein Atlas [24, 27]. Our study indicates that lung epithelia are NAPRT positive, but different types of brain cells may express NAPRT differently (eg neurons and oligodendrocytes are negative while reactive astrocytes are positive). Importantly, our results indicate that tumor infiltrating lymphocytes and other inflammatory cells stain NAPRT positive in the tumor samples examined, indicating that the niacin/NAMPTi combination could be compatible with immuno-oncology therapies such as the recently approved checkpoint inhibitors.

Further study on a larger population of cell samples will be needed to fully evaluate the status of NAPRT amongst different cell populations in the brain and other organs with the aim of identifying potential toxicities for the niacin/NAMPTi combination strategy, particularly for NAMPT inhibitors that penetrate the blood-brain-barrier. Together, these results present a novel and important theranostic antibody for the stratification of patients with NAPRT negative tumor tissue and identify some clinical indications that may benefit from therapy with NAMPTis in the presence of niacin.

## MATERIALS AND METHODS

### Cell culture

Cell lines H929, HT1080, H460, MIA PaCa-2, A549, MALME-3M, HCT-116, H1299, HT29, SW620, and SW480 were from the American Type Culture Collection (ATCC); U251 and DMS- 273 were from Sigma-Aldrich. All cell lines were cultured in RPMI 1640 medium (Wisent), supplemented with 10 % fetal bovine serum (FBS), 100 U/mL penicillin, 100 mg/mL streptomycin, and 2 mM L-glutamine, except MALME-3M which were cultured in McCoy's 5A medium, supplemented with 15 % FBS (all supplies from Wisent). Cells were incubated at 5 % CO_2_ and 37 °C under humidified conditions. All cell lines tested were mycoplasma negative by PCR (Charles River) prior to their experimental use.

### Recombinant protein expression

Full length *NAPRT* cDNA (representing amino acids 1-538) (Origene), and the region corresponding to the immunization fragment (amino acids 256-515) of *NAPRT* were cloned downstream of a GST tag into the pET151 vector (Invitrogen). Proteins were expressed in BL21(DE3) pLysS E. coli cells and recombinant NAPRT proteins purified using nickel-nitrilotriacetic acid chromatography (Qiagen), and concentrated using Amicon Ultra-15 centrifugal filter concentrators (Millipore). The GST tag was cleaved off of the immunization protein fragment using thrombin for use as a positive control protein in ELISA and immunoblot assays.

### Transfections

SiRNA knockdown in H1299 and H460 cells was performed in 6-well plates. SiRNA lipid complexes were allowed to form at room temperature for 30 minutes with 40 nM siRNA (OnTarget Plus Smartpool Cat# L-016912, Dharmacon/GE Healthcare) and 100 μL of RNAiMAX (Life Technologies) in 2 mL of OptiMEM. Cells (2.5 × 10^4^) in 2 mL of 20 % FBS and antibiotic free medium were added to the siRNA/lipid mixture for transfection (final siRNA concentration 20 nM). Plates were incubated at 37°C for 72 hours before cell harvest.

### Immunoblot analysis

Cells were lysed in buffer containing 20 mM Tris (pH 7.4), 100 mM NaCl, 1 % Triton X-100, 1 mM MgCl, 1 mM EDTA, 10 % glycerol, and supplemented with phosphatase and protease inhibitors (Sigma-Aldrich). Protein concentrations were determined using a Bio-Rad DC Protein Assay Kit (Bio-Rad). Proteins were separated by polyacrylamide gel electrophoresis (TGX Stain-Free – Precast Gels), and transferred to nitrocellulose membranes using the Trans-Blot Turbo Transfer System (Bio-Rad). Membranes were hybridized with primary antibodies against NAPRT: 40 ng/mL HPA023739 (Sigma-Aldrich), 100-200 ng/mL 3C6D2, 2 μg/mL CLO366, and 4 μg/mL each for antibodies 4A9 and 5B0 or anti-α-tulin antibody (DM1A, Sigma). Secondary antibodies were goat anti-mouse (1:10,000) or goat-anti rabbit (1:10,000) (Jackson ImmunoResearch). Detection of protein expression was assessed using the Western Lightning Plus-ECL detection Kit (PerkinElmer) and imaged with the ChemiDoc-Touch Imaging System (Bio-Rad).

### Cell viability assays

H1299 cells (1 -5 × 10^3^) were plated in 96 well plates in RPMI 1640 medium (Wisent), supplemented with 10 % FBS (Wisent), with no antibiotics. The following day, cells were treated with 50 nM GMX1778 (Gemin X Pharmaceuticals), with or without 10 μM nicotinic acid (Sigma-Aldrich). Plates were incubated for an additional 72 hrs at 37°C. Cell viability (measured by ATP levels) was quantified using CellTiter-Glo Luminescent Cell Viability Assay (Promega) according to manufacturer's instructions. Plates were read on a Pherastar FS (BMG Labtech).

### Monoclonal antibody production

Balb/c mice were immunized and boosted four times using a standard immunization protocol and purified NAPRT protein produced in E. coli (as described above). Antibody titers in sera from boosted mice were evaluated by ELISA and ICC. Once an acceptable titer was obtained, hybridoma fusion was performed using splenocytes from mice and the myeloma cell line sp2/0 [34]. Supernatants from ten different hybridoma clones were evaluated by ELISA and ICC. Two of the ten were selected for further sub-cloning by limiting dilution. The monoclonal antibody 3C6D2 was produced and purified from ascites. Immunizations, hybridoma creation and antibody production was carried out by ProMab Biotechnologies Inc.

### Tissue samples and immunohistochemistry assays

Tumor xenografts were grown by inoculating 1-5 × 10^6^ cells into Crl:NU(NCr)-*Foxn1^nu^* athymic nude mice (Charles River) and monitoring tumor cell growth until tumors reached 1000 mm^3^. Cell pellets or excised xenograft tumors were fixed in 10 % buffered formalin phosphate (Fisher Scientific) for 24 hrs at room temperature, followed by 70 % ethanol at room temperature, until the embedding process. Tissues were embedded in paraffin blocks, sliced into 5 μm thick sections, and then mounted onto glass slides (McGill University Histology Core Facility). Lung and brain tissue microarrays were obtained from (Montreal Neurological Institute - McGill University, USBioMAx, Cybrdi, and Origene). Tissue sections were de-paraffinized in Propar (Biocare Medical) and then rehydrated. Endogenous peroxidase was inactivated with 3 % hydrogen peroxide (Sigma), followed by heat induced epitope retrieval (HIER) using a NxGen Decloaking Chamber (Biocare Medical) and Borg Decloaker RTU solution (Biocare Medical). Slides were blocked for 15 min at room temperature with Background Sniper (Biocare Medical), and then incubated in 1-10 ng/mL monoclonal NAPRT antibody (3C6D2) or commercial antibodies HPA023739 (Sigma), CLO-366 (Atlas Cat# AMAb90725), 4A9 (LS-C139287, LSBio) and 5B0 (Creative Biomart) in Background Sniper (Biocare Medical) overnight at 4 °C. Peptide competitions were carried out with a 200-fold molar excess of Peptide 5-3-3, 5-3-4 or 5-3-1 (non-target control) or without competing peptide. Polymerization was performed using the MACH 1 Universal HRP polymer kit (Biocare Medical). 3-3-Diaminobenzidine (DAB) (Biocare Medical) was used for colorimetric detection. Tissue samples were counterstained with CAT haematoxylin (Biocare Medical), mounted with Eco-Mount (Biocare Medical), and photographed using a ScanScope Slide Scanner (Leica Biosystems). To evaluate NAPRT staining, the intensity of DAB was scored as either – (negative), + (positive), ++ (strongly positive), or +++ (very strongly positive) and a proportion of cells staining positive was determined for each sample.

### ELISA assays and epitope mapping

ELISA assays were carried out according to standard procedures using the NAPRT protein immunogen as a substrate in 50 mM Na_2_CO_3_-NaHCO_3_, 5.0 μg/mL, 100 μL/well, overnight, and blocked in 1 % BSA/PBS, 200 μL/well, 4 °C overnight. Serial dilutions of either mouse sera or hybridoma supernatant were incubated at 37 °C for 1 h followed by secondary antibody (HRP labeled anti-IgG; Sigma A0168) at: 1:9,000, 50 μL/well for 1 h at 37 °C. Colorimetric development occurred by adding 100 μL/well tetramethylbenzidine for 10 min at 37 °C followed by detection at 450 nm. The recombinant sequence of the NAPRT protein antigen was used for epitope mapping, 2 rounds of overlapping recombinant proteins, of decreasing size, and subsequent synthetic peptides were used to locate the epitope sequence being recognized by clone 3C6D2.

## SUPPLEMENTARY MATERIALS FIGURES



## References

[1] Houtkooper RH, Canto C, Wanders RJ, Auwerx J (2010). The secret life of NAD+: an old metabolite controlling new metabolic signaling pathways. Endocr Rev.

[2] Magni G, Orsomando G, Raffelli N, Ruggieri S (2008). Enzymology of mammalian NAD metabolism in health and disease. Front Biosci.

[3] D’Amours D, Desnoyers S, D’Silva I, Poirier GG (1999). Poly(ADP-ribosyl)ation reactions in the regulation of nuclear functions. Biochem J.

[4] Chiarugi A, Dolle C, Felici R, Ziegler M (2012). The NAD metabolome--a key determinant of cancer cell biology. Nat Rev Cancer.

[5] Kennedy BE, Sharif T, Martell E, Dai C, Kim Y, Lee PW, Gujar SA (2016). NAD+ salvage pathway in cancer metabolism and therapy. Pharmacol Res.

[6] Preyat N, Leo O (2016). Complex role of nicotinamide adenine dinucleotide in the regulation of programmed cell death pathways. Biochem Pharmacol.

[7] Samal B, Sun Y, Stearns G, Xie C, Suggs S, McNiece I (1994). Cloning and characterization of the cDNA encoding a novel human pre-B-cell colony-enhancing factor. Mol Cell Biol.

[8] Preiss J, Handler P (1958). Biosynthesis of diphosphopyridine nucleotide II. Enzymatic aspects. J Biol Chem.

[9] Niedel J, Dietrich LS (1973). Nicotinate phosphoribosyltransferase of human erythrocytes Purification and properties. J Biol Chem.

[10] Holen K, Saltz LB, Hollywood E, Burk K, Hanauske AR (2008). The pharmacokinetics, toxicities, and biologic effects of FK866, a nicotinamide adenine dinucleotide biosynthesis inhibitor. Invest New Drugs.

[11] von Heideman A, Berglund A, Larsson R, Nygren P (2010). Safety and efficacy of NAD depleting cancer drugs: results of a phase I clinical trial of CHS 828 and overview of published data. Cancer Chemother Pharmacol.

[12] Hovstadius P, Larsson R, Jonsson E, Skov T, Kissmeyer AM, Krasilnikoff K, Bergh J, Karlsson MO, Lonnebo A, Ahlgren J (2002). A Phase I study of CHS 828 in patients with solid tumor malignancy. Clin Cancer Res.

[13] Pishvaian MJ, Marshall JL, Hwang JH, Malik SM, He AR, Deeken JF, Kelso CB, Dorsch-Vogel K, Berger MS (2008). A phase 1 trial of GMX1777: An inhibitor of nicotinamide phosphoribosyl transferase (NAMPT). Journal of Clinical Oncology.

[14] Hegyi J, Schwartz RA, Hegyi V (2004). Pellagra: dermatitis, dementia, and diarrhea. Int J Dermatol.

[15] Roulston A, Shore G (2016). New strategies to maximize therapeutic opportunities for NAMPT inhibitors in oncology. Molecular & Cellular Oncology.

[16] Watson M, Roulston A, Belec L, Billot X, Marcellus R, Bedard D, Bernier C, Branchaud S, Chan H, Dairi K, Gilbert K, Goulet D, Gratton MO (2009). The small molecule GMX1778 is a potent inhibitor of NAD+ biosynthesis: strategy for enhanced therapy in nicotinic acid phosphoribosyltransferase 1-deficient tumors. Mol Cell Biol.

[17] Olesen UH, Thougaard AV, Jensen PB, Sehested M (2010). A preclinical study on the rescue of normal tissue by nicotinic acid in high-dose treatment with APO866, a specific nicotinamide phosphoribosyltransferase inhibitor. Mol Cancer Ther.

[18] Olesen UH, Hastrup N, Sehested M (2011). Expression patterns of nicotinamide phosphoribosyltransferase and nicotinic acid phosphoribosyltransferase in human malignant lymphomas. APMIS.

[19] Beauparlant P, Bedard D, Bernier C, Chan H, Gilbert K, Goulet D, Gratton MO, Lavoie M, Roulston A, Turcotte E, Watson M (2009). Preclinical development of the nicotinamide phosphoribosyl transferase inhibitor prodrug GMX1777. Anticancer Drugs.

[20] O’Brien T, Oeh J, Xiao Y, Liang X, Vanderbilt A, Qin A, Yang L, Lee LB, Ly J, Cosino E, LaCap JA, Ogasawara A, Williams S (2013). Supplementation of nicotinic acid with NAMPT inhibitors results in loss of in vivo efficacy in NAPRT1-deficient tumor models. Neoplasia.

[21] Xiao Y, Elkins K, Durieux JK, Lee L, Oeh J, Yang LX, Liang X, DelNagro C, Tremayne J, Kwong M, Liederer BM, Jackson PK, Belmont LD (2013). Dependence of tumor cell lines and patient-derived tumors on the NAD salvage pathway renders them sensitive to NAMPT inhibition with GNE-618. Neoplasia.

[22] Hasmann M, Schemainda I (2003). FK866, a highly specific noncompetitive inhibitor of nicotinamide phosphoribosyltransferase, represents a novel mechanism for induction of tumor cell apoptosis. Cancer Res.

[23] Shames DS, Elkins K, Walter K, Holcomb T, Du P, Mohl D, Xiao Y, Pham T, Haverty PM, Liederer B, Liang X, Yauch RL, O’Brien T (2013). Loss of NAPRT1 expression by tumor-specific promoter methylation provides a novel predictive biomarker for NAMPT inhibitors. Clin Cancer Res.

[24] Uhlen M, Fagerberg L, Hallstrom BM, Lindskog C, Oksvold P, Mardinoglu A, Sivertsson A, Kampf C, Sjostedt E, Asplund A, Olsson I, Edlund K, Lundberg E (2015). Proteomics. Tissue-based map of the human proteome. Science.

[25] Duarte-Pereira S, Silva SS, Azevedo L, Castro L, Amorim A, Silva RM (2014). NAMPT and NAPRT1: novel polymorphisms and distribution of variants between normal tissues and tumor samples. Sci Rep.

[26] Duarte-Pereira S, Pereira-Castro I, Silva SS, Correia MG, Neto C, da Costa LT, Amorim A, Silva RM (2016). Extensive regulation of nicotinate phosphoribosyltransferase (NAPRT) expression in human tissues and tumors. Oncotarget.

[27] Ponten F, Jirstrom K, Uhlen M (2008). The Human Protein Atlas--a tool for pathology. J Pathol.

[28] Olesen UH, Christensen MK, Björkling F, Jäättelä M, Jensen PB, Sehested M, Nielsen SJ (2008). Anticancer agent CHS-828 inhibits cellular synthesis of NAD. Biochem Biophys Res Commun.

[29] Marletta AS, Massarotti A, Orsomando G, Magni G, Rizzi M, Garavaglia S (2015). Crystal structure of human nicotinic acid phosphoribosyltransferase. FEBS Open Bio.

[30] Hara N, Yamada K, Shibata T, Osago H, Hashimoto T, Tsuchiya M (2007). Elevation of cellular NAD levels by nicotinic acid and involvement of nicotinic acid phosphoribosyltransferase in human cells. J Biol Chem.

[31] Galassi L, Di Stefano M, Brunetti L, Orsomando G, Amici A, Ruggieri S, Magni G (2012). Characterization of human nicotinate phosphoribosyltransferase: Kinetic studies, structure prediction and functional analysis by site-directed mutagenesis. Biochimie.

[32] Mehta S, Shelling A, Muthukaruppan A, Lasham A, Blenkiron C, Laking G, Print C (2010). Predictive and prognostic molecular markers for cancer medicine. Ther Adv Med Oncol.

[33] Tateishi K, Wakimoto H, Iafrate AJ, Tanaka S, Loebel F, Lelic N, Wiederschain D, Bedel O, Deng G, Zhang B, He T, Shi X, Gerszten RE (2015). Extreme Vulnerability of IDH1 Mutant Cancers to NAD+ Depletion. Cancer Cell.

[34] Shulman M, Wilde CD, Kohler G (1978). A better cell line for making hybridomas secreting specific antibodies. Nature.

