# Supplementary material for: Novel NAPRT specific antibody identifies small cell lung cancer and neuronal cancers as promising clinical indications for a NAMPT inhibitor/niacin co-administration strategy
Source: Oncotarget. 2017 Sep 12;8(44):77846–59. doi: 10.18632/oncotarget.20840 (PMC5652819; doi:10.18632/oncotarget.20840)
Supplement: Supplementary file 1 [file oncotarget-08-77846-s001.pdf]

Novel NAPRT specific antibody identifies small cell lung cancer and neuronal cancers as promising clinical indications for a NAMPT inhibitor/niacin co-administration strategy

SUPPLEMENTARY MATERIALS

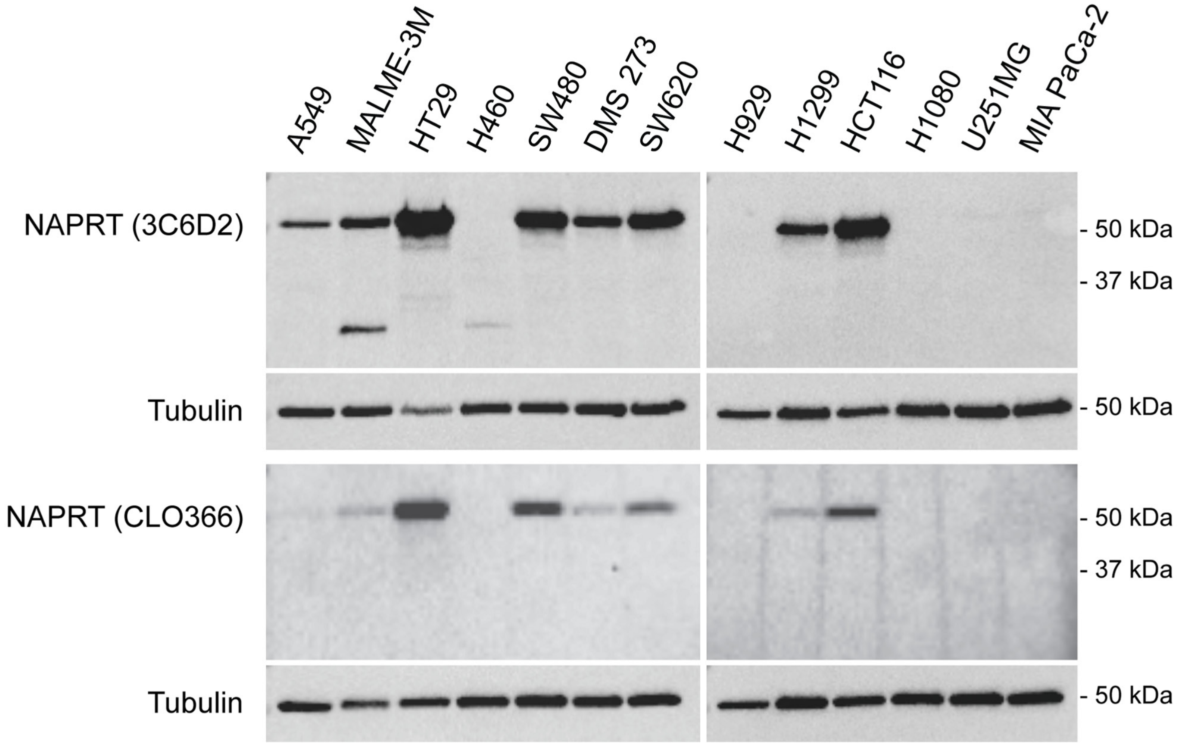

Supplementary Figure 1: Monoclonal antibody 3C6D2 has higher sensitivity than commercial antibody CLO366 by immunoblotting. NAPRT expression levels were determined in a panel of cancer cell lines by immunoblotting using monoclonal antibodies 3C6D2 (at 40 ng/mL) or CLO366 (at 2 µg/mL). Tubulin was used as a protein loading control.

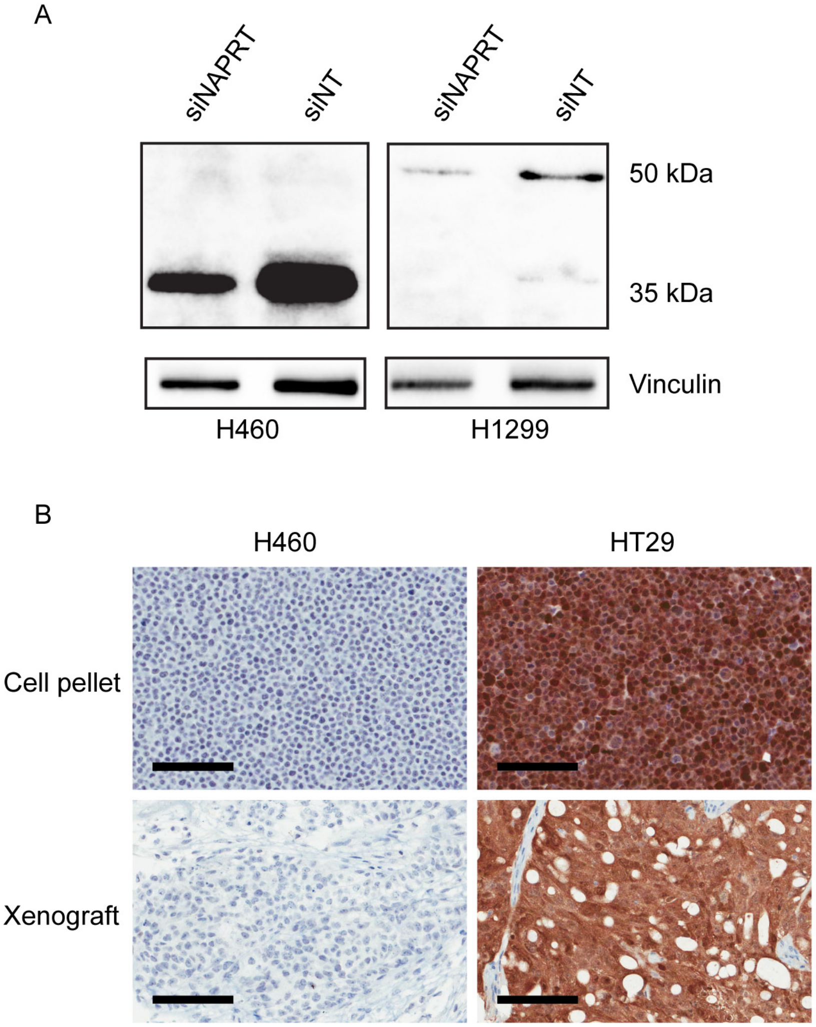

Supplementary Figure 2: H460 cells express a non-functional 35k Da form of NAPRT recognized by western blotting but not by IHC with 3C6D2. **(A)** H460 cells were transfected with a siRNA targeting NAPRT (siNAPRT), or a non-targeting siRNA (siNT) for 72 h, lysates were prepared and analysed by western blotting using the 3C6D2 monoclonal antibody. Vinculin was used as a loading control. **(B)** FFPE samples were prepared from either cell pellets of H460 or HT29 cells or from xenograft tumors grown in nude mice as indicated. Staining was performed using the 3C6D2 antibody; counter stain was with CAT hematoxylin. Scale bars, 100 µm

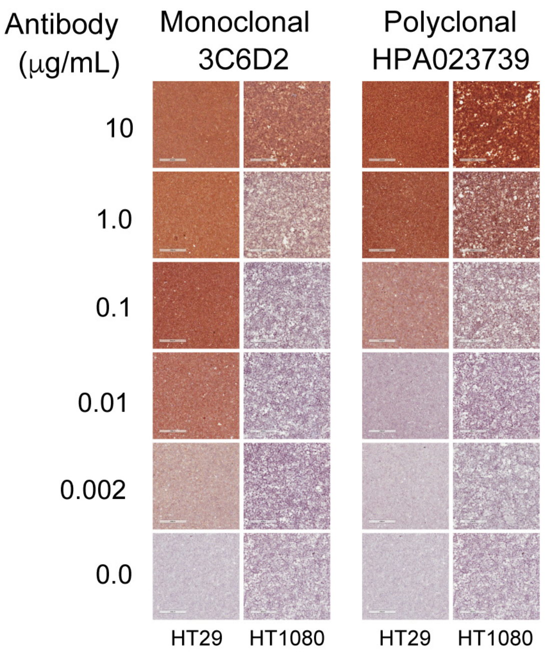

Supplementary Figure 3: Antibody 3C6D2 can distinguish NAPRT positive and negative cells at a greater concentration range by ICC than polyclonal antisera HPA023739. FFPE cell pellets from NAPRT positive HT29 and NAPRT negative HT1080 cells were stained with a concentration range of monoclonal 3C6D2 or the rabbit polyclonal antisera HPA023739 as indicated with CAT hematoxylin used as counter stain.
